# Supplementary figures and images for: Antibiotics alter development and gene expression in the model cnidarian Nematostella vectensis
Source: PeerJ. 2024 May 20;12:e17349. doi: 10.7717/peerj.17349 (PMC11114123; doi:10.7717/peerj.17349)

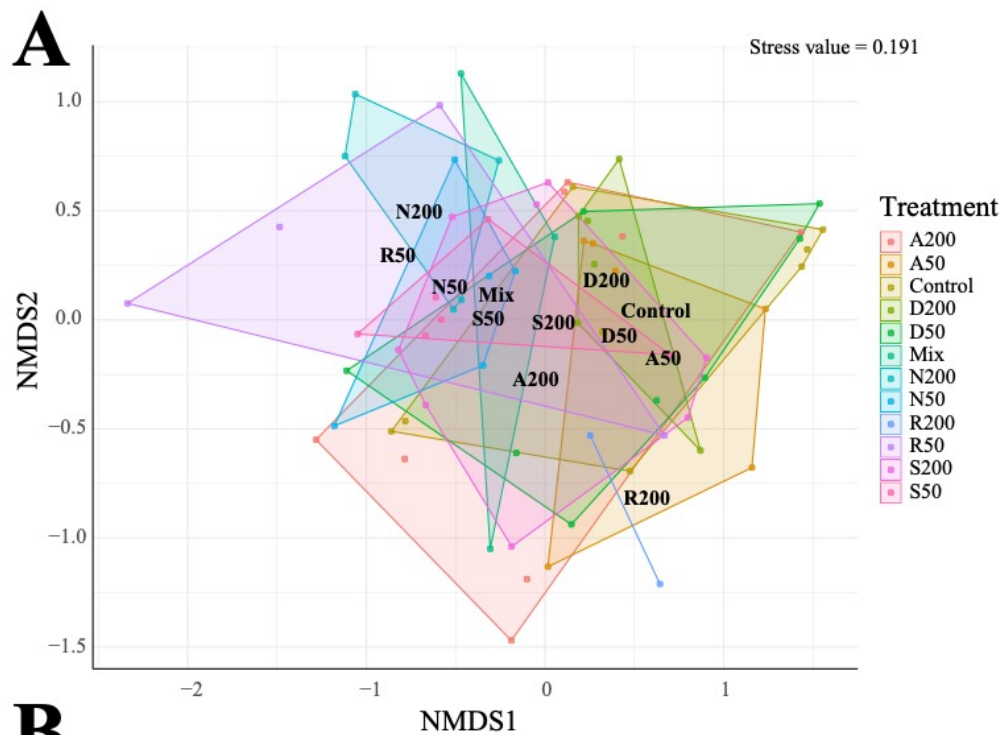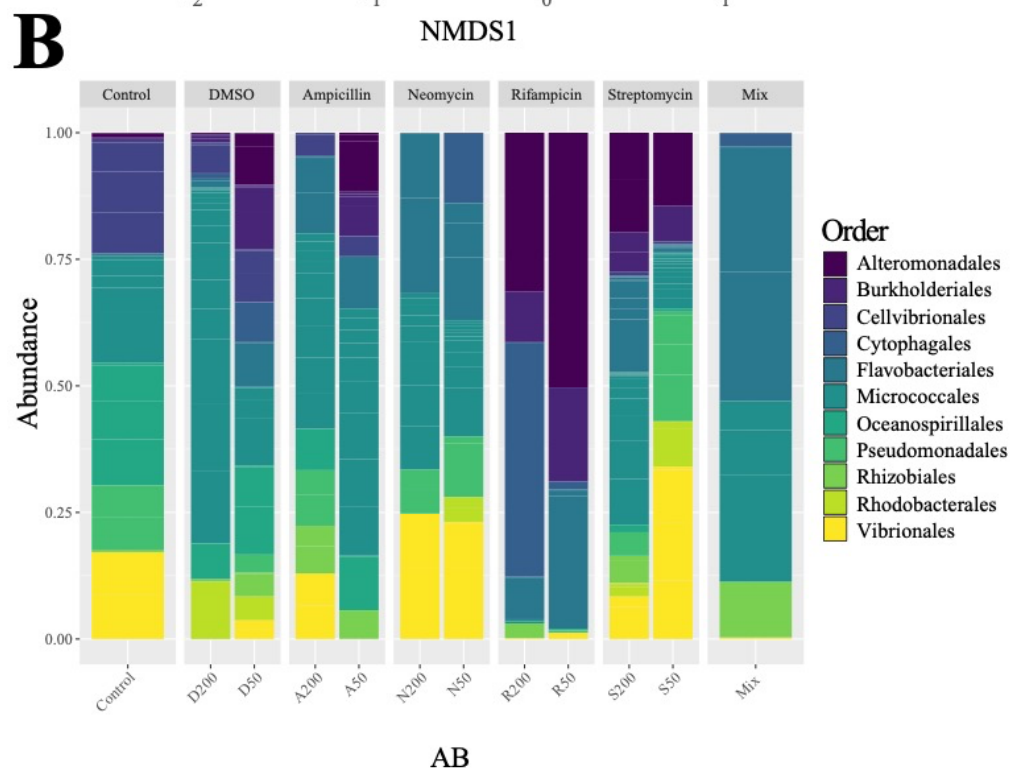

Supplement: Supplemental Information 1 — A) Non-metric multidimensional scaling plot, with a stress value of 0.191. (B) Abundance boxplots across conditions by rank Order. [file peerj-12-17349-s001.pdf]

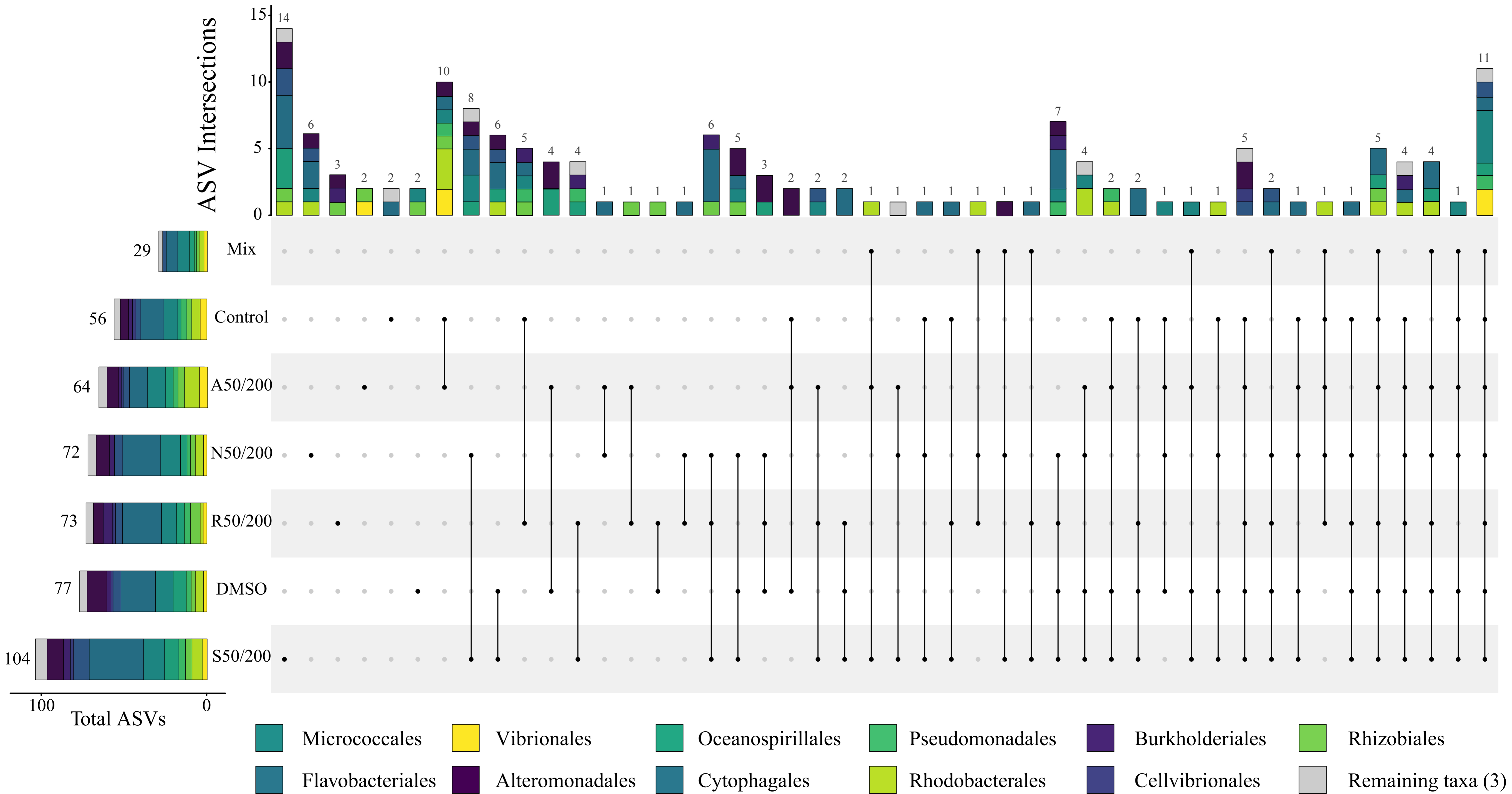

Supplement: Supplemental Information 2 — UpSet plot of individual ASVs per treatment. The vertical bars represent ASV intersections and the horizontal bars represent total ASVs per treatment. [file peerj-12-17349-s002.pdf]

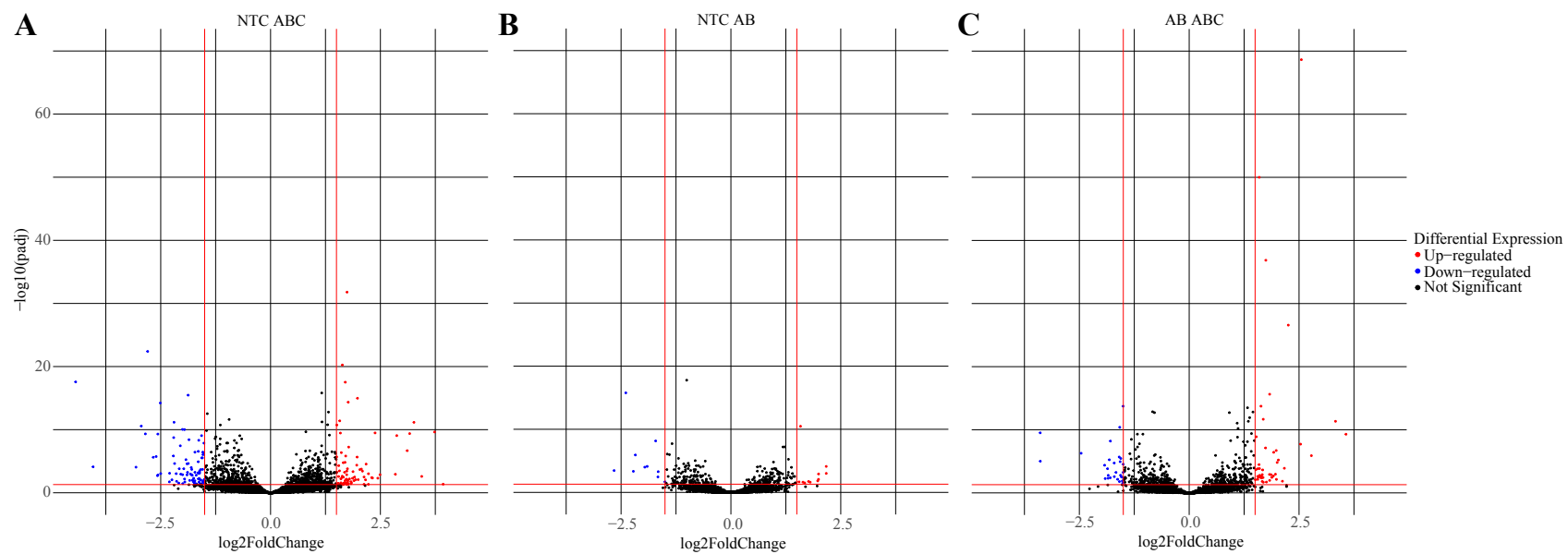

Supplement: Supplemental Information 3 — (A) Volcano plot NTC_ABA (B) Volcano plot NTC_ABC (C) Volcano plot ABA_ABC. The X-axis represents log fold base 2 change, and the Y-axis is the -log base 10 of the p adjusted values, and significance is calculated through +/- 1.5 log fold change and padj > 0.05. (NTC, No Treatment Control; AB, Antibiotic Acute; ABC, Antibiotic Constant). [file peerj-12-17349-s003.pdf]

## A - NTC AB BP

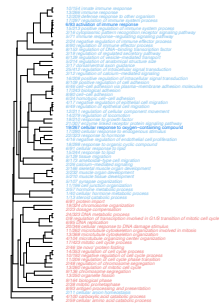

## B - NTC AB MF

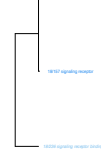

## C - NTC AB CC

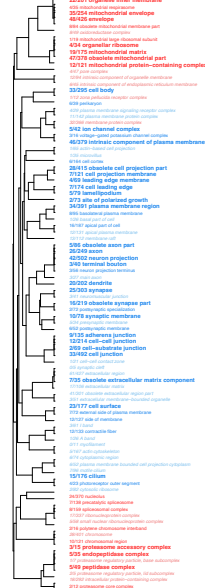

## D - NTC ABC MF

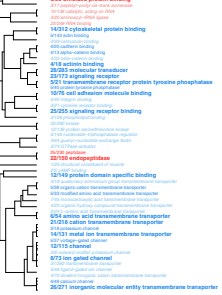

## E - NTC ABC CC

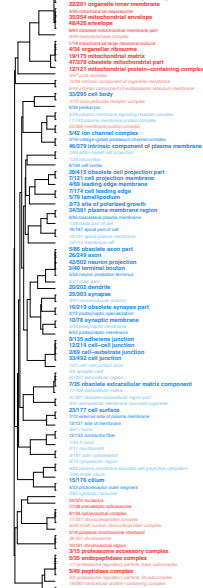

## F - NTC ABC BP

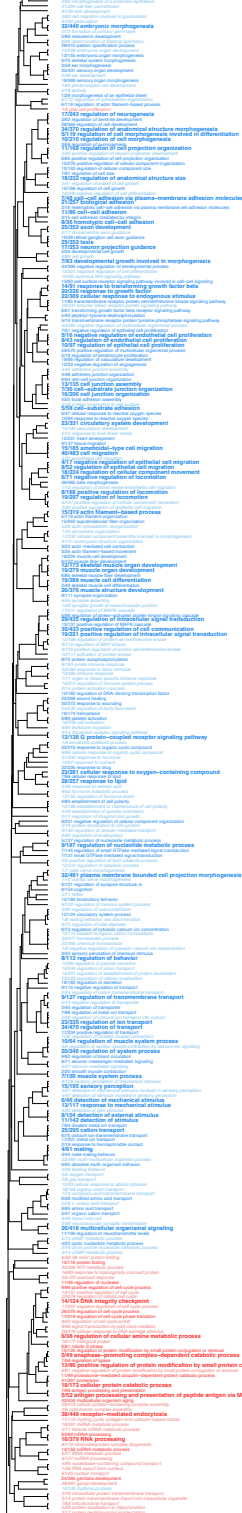

## G - AB ABC BP

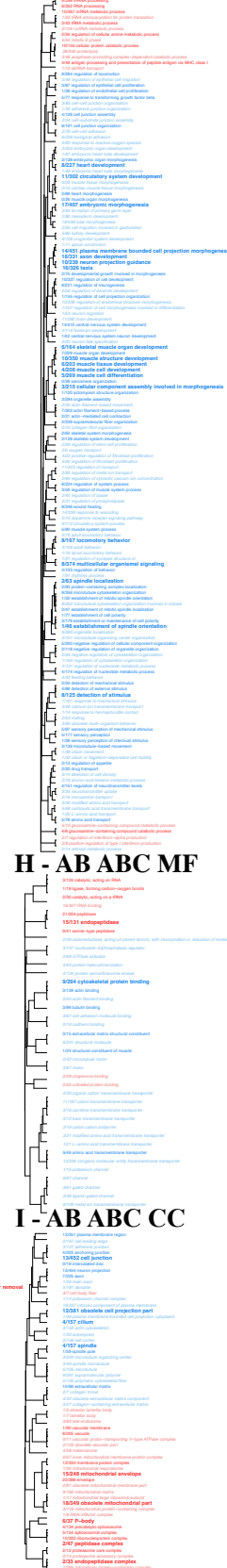

p < 0.001  
p < 0.01  
p < 0.05

## H - AB ABC MF

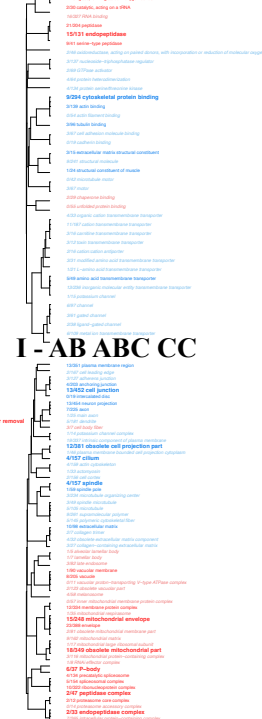

## I - AB ABC CC

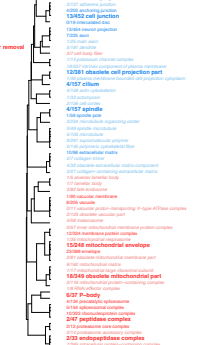

Supplement: Supplemental Information 4 — (NTC, No Treatment Control; AB, Antibiotic Acute; ABC, Antibiotic Constant; BP, Biological Process; MF, Molecular Function; CC, Cellular Component). [file peerj-12-17349-s004.pdf]
